# Luteolin Improves Cyclophosphamide-Induced Cystitis through TXNIP/NLRP3 and NF-*κ*B Pathways

**DOI:** 10.1155/2021/1718709

**Published:** 2021-11-11

**Authors:** Hengshuai Zhang, Jiang Zhao, Qudong Lu, Bishao Sun, Xin Liu, Chengfei Yang, Shuai Li, Longkun Li, Shanhong Yi, Zhenxing Yang, Jie Xu

**Affiliations:** Department of Urology, Second Affiliated Hospital, Army Medical University, Chongqing 400037, China

## Abstract

Hemorrhagic cystitis is an important complication of cyclophosphamide chemotherapy, and current therapies for the disease are limited. The natural flavonoid luteolin (LUT) has significant anti-inflammatory and antioxidant properties, but its protective effect on cyclophosphamide (CYP)-induced bladder toxicity has yet to be evaluated. This study aims to explore the protective effect of LUT on CYP-induced acute cystitis in rats. Female Sprague-Dawley rats were randomly assigned to the control (CON) group, CON + LUT group, CYP group, and CYP + LUT group. A single intraperitoneal injection of CYP was administered to establish an acute hemorrhagic cystitis model. HE staining was performed to detect the degree of bladder tissue damage, and TUNEL staining was performed to count apoptotic cells. Oxidative stress indicators were measured using commercial kits, and bladder surgery was performed to assess urinary function. The levels of inflammatory cytokines, apoptosis-related indicators, TXNIP/NLRP3 pathway, and NF-*κ*B pathway were detected by western blot. We found that LUT treatment reduced bladder bleeding, congestion, and edema caused by CYP. Compared with the CYP + LUT group, the level of apoptosis was more highly expressed in the CYP group. We also found that caspase-3, caspase-8, and Bax were significantly upregulated and Bcl-2 was downregulated after LUT treatment. In addition, LUT inhibited the activation of NF-*κ*B signal pathway in the rat bladder tissue after CYP exposure. LUT treatment can also reduce the NLRP3 inflammasome (NLRP3, ASC, and caspase-1) and TXNIP in the bladder. Finally, LUT can reduce the increase in the urination frequency and maximum urination pressure caused by cystitis. These results indicate that LUT displays effective anti-inflammatory, antioxidant, and antiapoptotic properties in CYP-induced acute hemorrhagic cystitis rats by inhibiting the TXNIP/NLRP3 and NF-*κ*B pathways. LUT may be a potent therapeutic agent for the prevention and treatment of hemorrhagic cystitis.

## 1. Introduction

Cyclophosphamide (CYP) is a commonly used drug for chemotherapy of tumors and autoimmune diseases, but it has side effects on organs such as the bladder, liver, and kidneys [1]. High-dose CYP often causes chemical cystitis, also known as hemorrhagic cystitis, which is specifically manifested as bladder mucosal damage, pelvic pain, and bladder dysfunction [2]. A recent study showed that nearly one-quarter of patients using high-dose CYP suffer from hemorrhagic cystitis [3]. The pathological mechanism of CYP-induced hemorrhagic cystitis is related to its metabolite acrolein. Acrolein is a product of CYP metabolism in the liver, and it accumulates in the bladder after being excreted by the kidneys. Acrolein can induce the production of reactive oxygen species (ROS) and nitric oxide (NO) to promote oxidative stress and inflammation and ultimately induce hemorrhagic cystitis through a variety of cascade reactions [4]. At present, mesna is the preferred drug for the prevention and treatment of hemorrhagic cystitis, but it is not ideal due to adverse reactions and instability of treatment [5]. Therefore, exploring new drugs for the prevention or treatment of hemorrhagic cystitis has important clinical significance.

Thioredoxin interacting protein (TXNIP) is a regulatory protein involved in oxidative stress [6], which can inhibit the thioredoxin (TRX) antioxidant system and participate in the occurrence of oxidative stress in many diseases such as ischemia-reperfusion injury [7], acute lung injury [8], and atherosclerosis [9]. Inhibition of TXNIP expression in ketamine-induced chemical cystitis can reverse apoptosis and oxidative stress [10]. TXNIP acts as a bridge between ROS and the NLRP3 inflammasome, linking oxidative stress and inflammation. Many studies have shown that the NOD-like receptor protein 3 (NLRP3) inflammasome is involved in the occurrence of bladder inflammation, and inhibiting the expression of NLRP3 can effectively improve bladder injury [11]. Nuclear factor-*κ*B (NF-*κ*B), as a proinflammatory transcription factor widely involved in a variety of inflammatory reactions, is activated during inflammation and amplifies the inflammatory response by regulating the release of proinflammatory factors such as TNF-*α* [12]. Activated NF-*κ*B has also been shown to increase the transcription of NLRP3 and promote the formation of inflammasome [13]. Therefore, TXNIP/NLRP3 and NF-*κ*B may be potentially effective targets for hemorrhagic cystitis.

**Figure 1:**
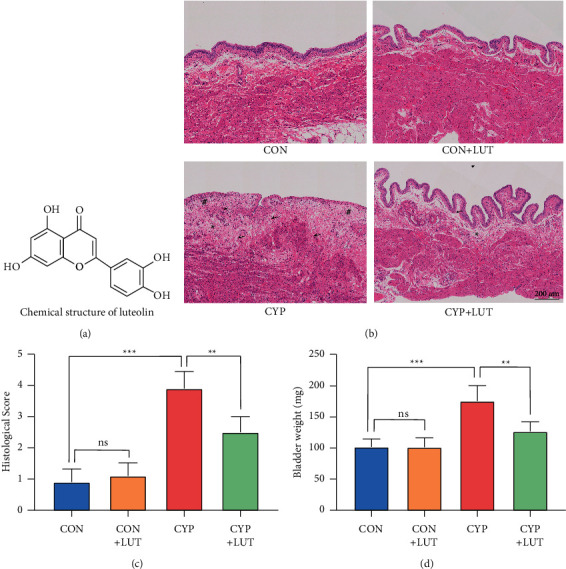
Effect of LUT on the pathological morphology of the bladder tissue in CYP-induced cystitis. (a) The chemical structure of LUT (3,4,5,7-hydroxyl-flavone). (b) Bladder microstructure from four groups of rats. In the CYP group, the bladder structure was severely damaged, with obvious bleeding, edema, and inflammatory cell infiltration. The shape of the bladder in the CYP + LUT group was close to normal. Arrows indicate bleeding, ∗ indicates edema, and # indicates infiltration of inflammatory cells. (c) Inflammation score under HE staining of the bladder in each group (*n* = 5). (d) The difference in the wet bladder weight between different groups (*n* = 6). ^*∗∗*^*P* < 0.01 and ^*∗∗∗*^*P* < 0.001; NS: not significant.

Luteolin (LUT) (Figure 1(a)) is a natural flavonoid compound widely found in honeysuckle, chrysanthemum, green pepper, celery, and other plants. LUT has many beneficial properties, including anti-inflammatory, antioxidant, wound-healing, nerve protection, and antitumor properties [14]. LUT has an excellent therapeutic effect on the chemical damage caused by the poison to the tissue. Recent studies have shown that LUT can reduce the damage caused by heavy metal cobalt to the hearts and kidneys of rats through its anti-inflammatory and antioxidant effects mediated by the NF-кB/Kim-1 pathway [15]. Wang et al. found that LUT can reduce inflammation, cell necrosis, and oxidative stress caused by lipopolysaccharide through the TXNIP-NLRP3 axis [16] and achieve a protective effect on the liver. In lower urinary tract diseases, LUT also shows a certain therapeutic effect. According to reports, luteolin can improve bladder dysfunction caused by diabetic bladder disease by downregulating SCF/c-kit and PI3K [17]. LUT can also protect the bladder epithelium from *E. coli* infection by inhibiting cAMP-phosphodiesterase [18]. The therapeutic effect of LUT on chemical toxicity injury and lower urinary tract disease indicates that it may have a protective effect on CYP-induced hemorrhagic cystitis. Therefore, this study aims to verify the therapeutic effect of LUT on acute hemorrhagic cystitis in rats and to further determine whether the NF-*κ*B signal pathway and the TXNIP/NLRP3 axis are the therapeutic targets of LUT against bladder injury.

## 2. Materials and Methods

### 2.1. Animals

The female SD rats (200–240 g, 10–16 weeks old) used in this experiment were purchased from the Experimental Animal Center of Army Medical University. The experimental animals were kept at 20–23°C in a standard environment with the same length of day and night, and they were given unlimited food and water. All animal research was authorized by the Laboratory Animal Welfare and Ethics Committee of the Army Military Medical University (project identification code: AMUWEC2019416), and the study complied with the Animal Welfare Guidelines and the Declaration of Helsinki.

### 2.2. Animal Groupings and Administered Treatments

Before the experiment, the rats were randomly divided into the CON (control), CON + LUT, CYP, and CYP + LUT groups. During the one-week experimental period, the CON + LUT group and CYP + LUT group were given LUT 100 mg/kg (T2682, TCI, Japan) via daily gavage for 7 days [19]. The CON group and CYP group were treated with an equivalent volume of saline by gavage. On the fifth day of the experiment, the CYP group and CYP + LUT group were injected with CYP (150 mg/kg) (HY17420, MCE, USA) by intraperitoneal injection to induce acute hemorrhagic cystitis [20], and the CON group and CON + LUT group received normal saline solution intraperitoneally. The experiment was terminated on day 7. Some rats were sacrificed for tissue collection, and the remaining rats were used for continuous cystometry experiments.

### 2.3. Tissue Sections and HE Staining

The bladder tissue was collected through a midline incision in the lower abdomen 48 hours after intraperitoneal injection of CYP or saline. The harvested bladder was divided into two parts longitudinally; one part was used for histological research, and the other part was used for western blotting or oxidative stress measurement. The fixed bladder was cut into 5 *μ*m thick sections and adhered to a glass slide. The hematoxylin-eosin (HE) staining was carried out as described previously [21]. Four visual fields (400×) were randomly selected from the HE stained sections, and the inflammation score was measured according to the standards reported in the literature [22].

### 2.4. TUNEL Staining

According to the manufacturer's protocol, bladder sections were analyzed by TUNEL assay using a Tunel Cell Apoptosis Detection Kit (G1507, Servicebio, China). In brief, bladder sections were deparaffinized and permeabilized with proteinase K. Membrane breaking working liquid was then used to rupture the membrane. PBS containing 0.3% hydrogen peroxide was used to block endogenous peroxidase for 30 minutes. After washing three times with PBS, the bladder sections were dripped with recombinant TdT enzyme and incubated in a 37°C incubator for 1 hour for the end labeling reaction. After washing again, the bladder sections were treated with streptavidin-HRP reaction solution and incubated at 37°C for 30 min. The DAB solution was added to the section, and the color development was monitored under an optical microscope. The reaction was stopped by washing with deionized water for 3 min. Finally, bladder sections were counterstained with hematoxylin, dehydrated, and mounted. Five fields in the TUNEL-stained section were randomly selected under a 400X microscope to determine the apoptotic index (the proportion of apoptotic cells in the total cells).

### 2.5. Estimation of Bladder Oxidative Stress Indicators

The harvested bladder was treated with physiological saline at a ratio of 1 : 10 and then polished into a homogenate. The tissue homogenate was centrifuged at 3000 rpm for 12 minutes, and the supernatant was extracted. According to the manufacturer's instructions, the levels of malondialdehyde (MDA), total superoxide dismutase (SOD), and glutathione (GSH) in the bladder were measured using commercial kits (A003, A001, and A006) obtained from the Nanjing Jiancheng Institute of Bioengineering.

### 2.6. Western Blot

The protein in the bladder tissue was extracted with RIPA buffer, and its concentration was measured with BCA Protein Assay Reagent (P0012, Beyotime, China). 30 *μ*g of protein in each sample was loaded onto the SDS-PAGE gel for electrophoresis and then transferred to the PVDF membranes (ISEQ00010, Merck Millipore, Germany). After being blocked for one hour, the membranes were incubated overnight at 4°C with the following primary antibodies: NLRP3 (1 : 500, ab270449, Abcam, UK), TXNIP (1 : 1000, ab188865, Abcam, UK), Bcl-2 (1 : 1000, ab196495, Abcam, UK), IL-6 (1 : 500, P620, Thermo Fisher, USA), p–NF–*κ*B (1 : 1000, 3033, CST, USA), NF-*κ*B (1 : 1000, 8242, CST, USA), IL-1*β* (1 : 1000, sc12742, Santa, USA), caspase-3 (1 : 1000, 66470, Proteintech, China), caspase-8 (1 : 1000, 13426, Proteintech, China), Bax (1 : 1000, 60267, Proteintech, China), TNF-*α* (1 : 1000, 17590, Proteintech, China), TRX (1 : 1000, 14999, Proteintech, China), I*κ*B*α* (1 : 1000, 4814, CST, USA), p-I*κ*B*α* (1 : 1000, 2859, CST, USA), caspase-1(1 : 1000, 22915, Proteintech, China), ASC (1 : 1000, sc514414, Santa, USA), and GAPDH (1 : 1000, 60004, Proteintech, China). The membranes were rinsed in TBST the next day before being incubated with goat anti-rabbit secondary antibody (1 : 2000, G6120, Thermo Fisher, USA) or goat anti-mouse secondary antibody (1 : 2000, G21040, Thermo Fisher, USA). Finally, ECL Substrate (VK312464, Thermo Fisher, USA) and a bioanalytical imaging system (C300, Azure Biosystems, USA) were used to detect protein bands.

### 2.7. Continuous Cystometry

Five rats in each group were taken for cystometry measurement to evaluate the urodynamic contraction pressure and time as previously described [23]. In brief, rat bladders were exposed through an abdominal incision after they were anesthetized with urethane. Then, a tiny incision was made on the top of the bladder; the PE-50 catheter was connected to the bladder cavity and secured with sutures. The tube was connected to a microperfusion pump (YPJ01, Smiths Medical, USA) and received saline infusion (10 ml/h). The bladder function reached a stable state about 30 minutes after the start of the experiment. At this time, the urination waveform data were recorded using a data acquisition system (RM6240, Chengdu Instrument Factory, China), and the maximum bladder pressure (MBP) and intercontractile interval (ICI) between the different groups were measured and compared.

### 2.8. Statistical Analysis

Statistical analysis was performed using SPSS statistics 26 (SPSS Inc, USA). All data are presented as the mean ± standard error of the mean (mean ± SEM). The histological scores and apoptotic index scores were statistically compared between groups by Kruskal–Wallis multiple comparison tests. The other data were assessed with two-way ANOVA, and Bonferroni correction was followed. *P* < 0.05 indicated statistical significance (NS: not significant; ^*∗*^*P* < 0.05, ^*∗∗*^*P* < 0.01, and ^*∗∗∗*^*P* < 0.001).

## 3. Results

### 3.1. LUT Treatment Improves the Histopathology of CYP-Induced Cystitis

We first performed a histological examination of the bladder to preliminarily judge the therapeutic effect of LUT. The bladder structure was normal in the CON group and CON + LUT group. The epithelial structure was complete without obvious pathological changes. In the CYP group, the bladder mucosa and lamina propria showed obvious congestion, bleeding, edema, ulcers, and inflammatory cell infiltration 48 hours after the induction of cystitis. LUT treatment reduced the pathological damage caused by CYP to the bladder tissue, and the phenomenon of edema and bleeding was improved (Figure 1(b)). The histological score also showed that the degree of tissue damage in the CYP + LUT group was better than that in the CYP group (Figure 1(c)). A general examination of the bladder revealed that CYP caused a significant increase in wet bladder weight, while LUT improved bladder edema and reduced bladder weight (Figure 1(d)).

### 3.2. Effect of LUT on Oxidative Stress Indexes and Inflammatory Cytokine Levels in CYP-Induced Cystitis

**Figure 2:**
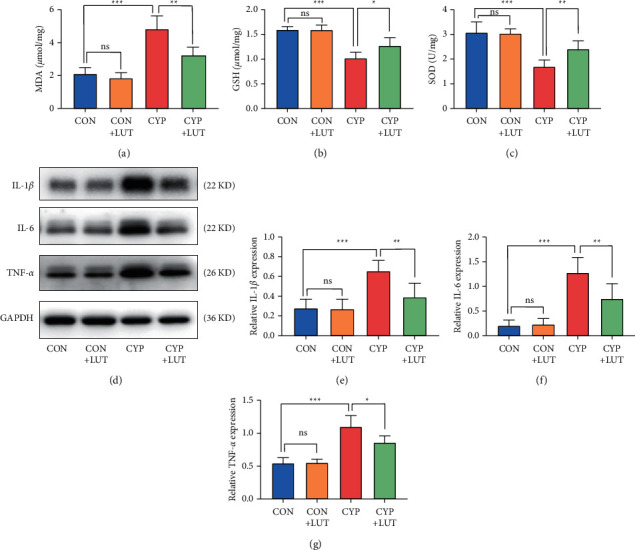
LUT inhibits the increase in oxidative stress and inflammatory cytokine levels in the bladder tissue caused by CYP. The measurement results of MDA (a), GSH (b), and SOD (c) levels in the bladder of each group. LUT treatment reduced the disorder of bladder oxidative stress-related molecules caused by CYP. (d) The expressions of IL-1*β*, IL-6, and TNF-*α* in the bladder of four groups were detected by western blot. LUT inhibited the upregulation of inflammatory factors induced by CYP. The expression analysis of IL-1*β* (e), IL-6 (f), and TNF-*α* (g) protein in the bladder of four groups (*n* = 6). *∗P* < 0.05, ^*∗∗*^*P* < 0.01, and ^*∗∗∗*^*P* < 0.001; NS: not significant.

To further clarify the potential mechanism of LUT, we measured GSH, SOD, and MDA levels in the bladder to assess the level of bladder oxidative stress. Compared with the CON group, there was no significant difference in the abovementioned indicators in the CON + LUT group. Compared with those of normal rats, the activities of SOD and GSH in the CYP group were significantly reduced and the level of MDA was significantly increased. However, LUT treatment inhibited the increase of MDA in the bladder and partially restored GSH and SOD activity (Figures 2(a)–2(c)). In addition, we measured bladder proinflammatory cytokines with western blot. The results showed that the levels of IL-6, IL-1*β*, and TNF-*α* in the bladder of the CYP group were significantly increased. LUT treatment significantly reduced the inflammatory cytokines, while the expression of these inflammatory proteins did not change in normal rats after using LUT (Figure 2(d)).

### 3.3. LUT Decreases the Apoptosis Level in CYP-Induced Cystitis

**Figure 3:**
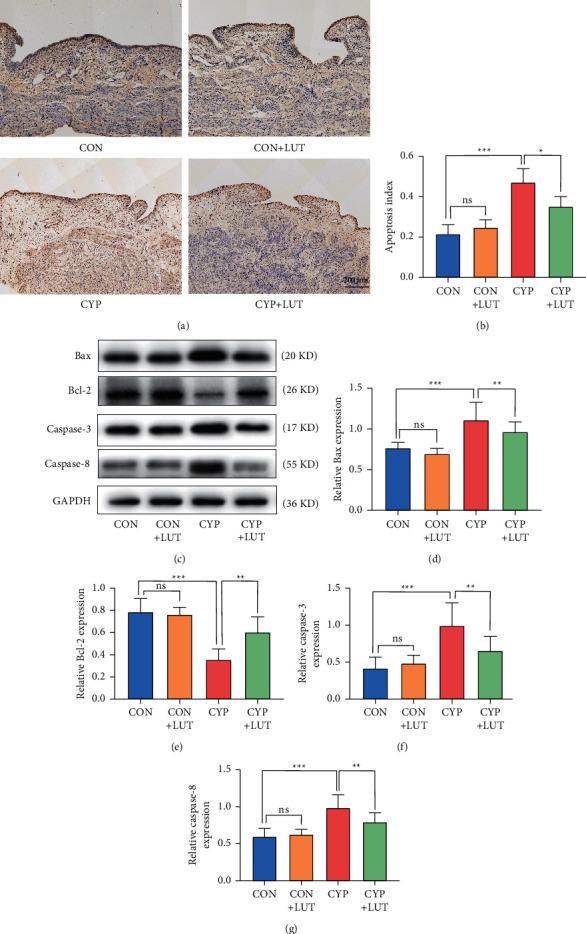
LUT reduces apoptosis in CYP-induced cystitis. (a) TUNEL staining of bladder slices in each group. Apoptotic cells were stained dark brown and nonapoptotic cells were stained blue. (b) Statistics of apoptotic index from TUNEL staining in each group (*n* = 5). LUT reduced the number of TUNEL staining positive cells in the bladder. (c) The expression of Bax, Bcl-2, caspase-3, and caspase-8 in the bladder of each group was detected by western blot. LUT treatment ameliorated the disorder of apoptosis-related proteins in CYP-induced cystitis. The expression analysis of Bax (d), Bcl-2 (e), caspase-3 (f), and caspase-8 (g) proteins in the bladder of each group (*n* = 6). ^∗^*P* < 0.05, ^*∗∗*^*P* < 0.01, and ^*∗∗∗*^*P* < 0.001; NS: not significant.

Subsequently, we further detected the apoptosis level of bladder tissue by TUNEL staining and western blotting. Apoptosis was not obvious in the CON group and the CON + LUT group, while the number of apoptotic cells increased significantly in the CYP group, especially in the bladder mucosa. However, LUT treatment reduced the high levels of apoptosis induced by CYP (Figures 3(a) and 3(b)). Western blot results showed that the expression of Bax, caspase-3, and caspase-8 in the bladder of the CYP group was significantly upregulated while Bcl-2 was inhibited. Compared with the CYP group, the disorder of these apoptosis-related proteins in the CYP + LUT group was significantly improved (Figures 3(c)–3(g)), further proving that LUT can reduce CYP-induced bladder cell apoptosis.

### 3.4. Effect of LUT on the TXNIP/NLRP3 Axis in CYP-Induced Cystitis

**Figure 4:**
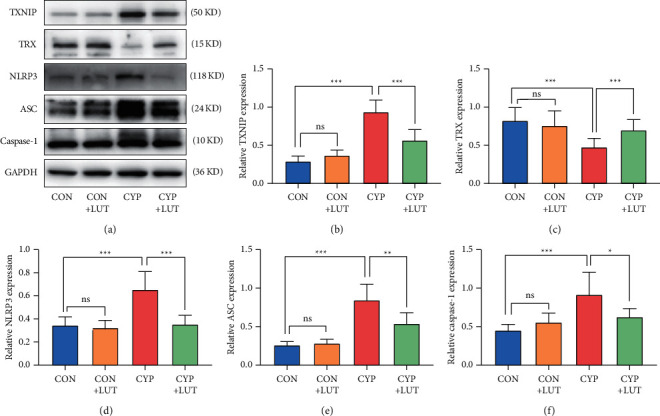
LUT inhibits the TXNIP/NLRP3 pathway in CYP-induced cystitis. (a) The expression of TXNIP, TRX, NLRP3, ASC, and caspase-1 in each test group was detected by western blot. LUT inhibited the upregulation of TXNIP, NLRP3, ASC, and caspase-1 in CYP-induced cystitis and increased the expression of TRX. The expression analysis of TXNIP (b), TRX (c), NLRP3 (d), ASC (e), and caspase-1 (f) protein in the bladder of each group (*n* = 6). ^∗^*P* < 0.05, ^*∗∗*^*P* < 0.01, and ^*∗∗∗*^*P* < 0.001; NS: not significant.

The regulating effect of the TXNIP/NLRP3 axis on oxidative stress has been widely reported [16]. In order to study the mechanism of LUT improving CYP-induced cystitis, we used western blot to study the expression changes of TXNIP/NLRP3 before and after LUT treatment.  Figure 4 shows that the expression of TXNIP, NLRP3, ASC, and caspase-1 protein in the CYP group was significantly increased compared to those of the CON group. However, the expression levels of TXNIP, NLRP3, ASC, and caspase-1 proteins in the CYP + LUT group decreased, and the expression of TRX increased. In conclusion, our results indicate that the activation of the TXNIP/NLRP3 pathway was suppressed by LUT.

### 3.5. LUT Inhibits the Activation of NF-*κ*B Signal Pathway Caused by CYP

**Figure 5:**
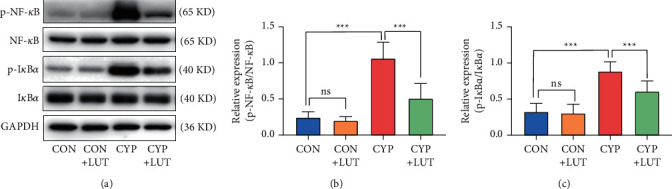
LUT inactivates the NF-*κ*B pathway in CYP-induced cystitis. (a) The expression of p-NF-*κ*B, NF-*κ*B, p-I*κ*B, and I*κ*B in the bladder of each group was detected by western blot. LUT inhibited the phosphorylation of NF-*κ*B and I*κ*B*α* in the bladder caused by CYP. Analysis of the levels of NF-*κ*B (b) phosphorylation and I*κ*B*α* (c) phosphorylation in each group (*n* = 6). ^*∗∗∗*^*P* < 0.001; NS: not significant.

The role of inflammation in CYP-induced cystitis cannot be ignored. We further studied the changes of the inflammatory NF-*κ*B pathway in these four groups. The expression of p-I*κ*B*α* and p-NF-*κ*B in the CYP group was significantly increased compared with that of the CON group (Figure 5). The LUT treatment inhibited the phosphorylation of NF-*κ*B and I*κ*B*α*.

### 3.6. LUT Improves Bladder Dysfunction in Hemorrhagic Cystitis

**Figure 6:**
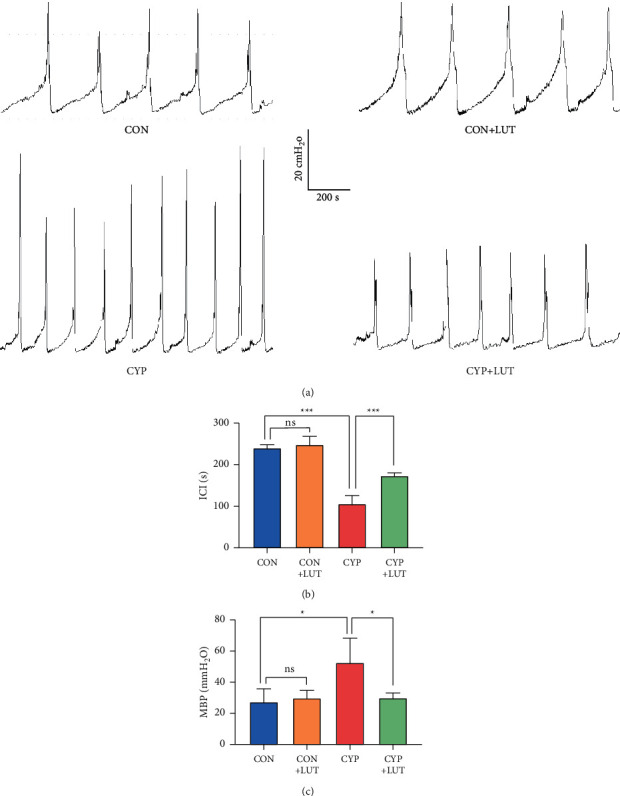
LUT improves bladder dysfunction induced by CYP. (a) Pressure waveform during urination of the CON group, CON + LUT group, CYP group, and CYP + LUT group. LUT improves abnormal urination patterns caused by CYP. Comparison of maximum bladder pressure (b) and intercontractile interval (c) in each group (*n* = 5). ^∗^*P* < 0.05 and ^*∗∗∗*^*P* < 0.001; NS: not significant.

To evaluate the effect of LUT treatment on bladder function, we performed cystometry to measure the bladder voiding time and contraction of the four groups of rats. The maximum bladder pressure (MBP) of the CYP group was higher than that of normal rats, and the pressure showed greater fluctuation. The MBP of rats treated with LUT decreased significantly, and the urination pressure was more stable (Figures 6(a) and 6(b)). In addition, we observed that CYP shortened intercontractile intervals (ICIs), corresponding to an increase in the urination frequency. The ICI of the LUT + CYP group was significantly increased, but it was shorter than that of the CON group and the CON + LUT group (Figure 6(c)).

## 4. Discussion

Hemorrhagic cystitis, as a serious complication caused by the chemotherapy drug CYP, has brought great trouble to the use of CYP in tumor and rheumatic diseases. Preventive use of urinary tract protectors is currently the main method to solve this problem, but the search for a more effective and safer treatment of hemorrhagic cystitis is still widely underway [5]. LUT is a natural flavonoid compound found in many edible plants. It has been shown to play a protective role in a variety of diseases, such as acute lung injury caused by sepsis [8] and myocardial ischemia-reperfusion injury [24]. In this study, we verified the protective effect of LUT on CYP-induced cystitis. We found that LUT treatment can inhibit the level of bladder inflammation, oxidative stress, and apoptosis. In addition, we found that LUT may mediate anti-inflammatory and antioxidant effects by inhibiting NK-*κ*B activity and TXNIP/NLRP3 axis activation. Finally, the urodynamic parameters we collected proved that LUT treatment improved the abnormal bladder function caused by hemorrhagic cystitis.

The typical pathological manifestation of hemorrhagic cystitis includes edema, ulcers, submucosal hemorrhage, epithelial injury, and inflammatory cell infiltration [25]. Consistently, we found that CYP-treated rats showed these typical characteristics. The increase in bladder weight may be due to inflammation damage, which leads to increased microvascular permeability and thus to tissue edema. Cystometry is an effective method to assess bladder function. Previous studies have found that bladder compliance decreases and urination frequency increases after CYP treatment [26]. Similarly, bladder neuropathy caused by diabetes has been shown to increase the maximum bladder capacity and residual urine volume. LUT restores bladder function by inhibiting the activity of SCF and c-Kit [17]. In our study, LUT treatment significantly improved the pathological damage and urination abnormalities caused by CYP, proving the protective effect of LUT on the bladder.

It is generally believed that oxidative stress plays a leading role in the pathogenesis of CYP-induced cystitis [27]. Under normal physiological conditions, the antioxidant system composed of SOD and GSH can promptly remove ROS and other free radicals generated by oxidation reactions and maintain a balanced state [28]. A large amount of ROS induced by acrolein disrupts the oxidative balance of the bladder epithelium, further leads to the destruction of DNA structure, and induces lipid peroxidation to form MDA [29], accompanied by a decrease in GSH and SOD levels [30]. As an excellent antioxidant, LUT can effectively reduce the ROS production of macrophages under the stimulation of LPS [31]. Also, in a study of a mouse model of liver failure, LUT treatment increased the glutathione content of the tissues and decreased lipid peroxidation [32]. Our results also prove that LUT treatment can effectively improve the disorder of bladder oxidative stress indicators. Therefore, it can be concluded that LUT improves hemorrhagic cystitis by maintaining SOD and GSH activity and suppressing MDA production.

As we all know, TXNIP and TRX are important components in the thioredoxin system that regulates oxidative stress in the body [33]. TRX is a key antioxidant protein that exists in the cytoplasm to protect proteins and nucleic acids from oxidative damage. TXNIP, as a negative regulator of TRX, increases the sensitivity of tissues to oxidative stress [6]. The occurrence of oxidative damage in ketamine-induced cystitis resulted in high expression of TXNIP in the bladder [10], and consistent results were also found in CYP-induced cystitis. In addition, we also observed a significant decrease in the expression of TRX, which may be related to the inhibitory effect of TXNIP on TRX and the inhibitory effect of cyclophosphamide on the activity of thioredoxin reductase [34]. ROS leads to the dissociation of TXNIP from the TXNIP/TRX complex. Subsequently, TXNIP binds to NLRP3 and promotes the activation of inflammasome [35]. It has been proved that abnormal or excessive activation of NLRP3 contributes to the development of bladder inflammation [36]. Recent studies have shown that the use of drugs to inhibit the activation of NLRP3 alleviates the pyrolysis in acute interstitial cystitis [11]. Our results showed that LUT treatment significantly inhibited the expression of TXNIP and the NLRP3 inflammasome constituent proteins (NLRP3, caspase-1, and ASC). This is consistent with similar results by Wang et al. [16]. This illustrates that LUT exerts bladder-protective effects by inhibiting the TXNIP/NLRP3 axis.

Tissue damage caused by oxidative stress is usually accompanied by inflammation; the NF-*κ*B signal is the main signal pathway that mediates the production of inflammatory factors [12]. Under normal conditions, NF-*κ*B usually binds to its inhibitor I*κ*B*α* in the form of dimers and is in an inactive state. The oxidative stress induced by CYP leads to the activation of I*κ*B kinases (IKKs) and the degradation and phosphorylation of I*κ*B*α*. At the same time, NF-*κ*B dissociates and transfers to the nucleus to induce the production of inflammatory factors [12]. Increasing inflammatory mediators such as TNF-*α*, IL-6, and IL-1*β* in hemorrhagic cystitis will further aggravate the inflammatory response [37]. According to reports, LUT can prevent the activation of NF-*κ*B in the colon tissue stimulated by ammonium thiosulfate through the HMGB1-TLR pathway [38]. In this study, LUT can inhibit the activation of the NF-*κ*B signal induced by CYP. In addition, the increased inflammatory factors such as TNF-*α* caused by CYP were reversed by LUT treatment. This illustrates that LUT exerts anti-inflammatory effects by inhibiting the NF-*κ*B pathway.

Apoptosis is the key mechanism of CYP-induced bladder toxicity [39], which can be triggered by inflammatory factors and oxidative stress [40]. Chronic intermittent hypoxia-induced mitochondrial dysfunction mediates endothelial injury via the TXNIP/NLRP3/IL-1*β* pathway. Antiapoptotic Bcl-2 can inhibit the permeability changes of the outer mitochondrial membrane caused by Bax to prevent the release of cytochrome C into the cytoplasm, thereby inhibiting the occurrence of apoptosis [41]. In previous studies, it was found that CYP can cause urothelial cell apoptosis by upregulating the ratio of Bax/Bcl-2 [42]. Caspase-8 participates in apoptosis induced by the death receptor pathway [43]. As a key mediator of the executive function of the terminal stage of apoptosis, caspase-3 can be activated by death receptors and mitochondrial pathways to induce the occurrence of apoptosis [44]. In previous studies, it was found that LUT treatment can counteract cell apoptosis, which is achieved by regulating the apoptosis markers Bcl-2, Bax, and caspase-3 [45]. In addition, the caspase inhibitory effect of LUT may be related to its antiapoptotic effect [46]. In accordance with these reports, our findings revealed that LUT therapy can improve bladder injury in rats with hemorrhagic cystitis by controlling cell apoptosis.

Previous studies have shown that the TXNIP/NLRP3/IL-1*β* pathway is involved in cell apoptosis under hypoxia. In addition, the knockdown of NLRP3 can protect cells from apoptosis [47]. This finding indicates that LUT can inhibit CYP-induced bladder cell apoptosis through the TXNIP/NLRP3 axis. NF-*κ*B activation is also involved in the initiation of apoptosis in inflammatory diseases [48]. Existing reports have shown that the nuclear translocation of NF-*κ*B and the significant increase in Bax expression in the bladder tissue are related to bladder cell apoptosis [48]. NF-*κ*B activation mediates the process of oxidative stress-induced apoptosis by reducing Bcl-2 protein activation and Bax protein translocation and enhancing p53 function [49]. NF-*κ*B also promotes apoptosis by upregulating the expression of death receptors [50]. Although the effect of NF-*κ*B is not direct, our results also indicate that luteolin can protect the bladder from apoptosis and inflammation by inhibiting NF-*κ*B.

## 5. Conclusions

In summary, we found that LUT can inhibit oxidative stress, inflammation, and apoptosis through TXNIP/NLRP3 and NF-*κ*B, which then produces protection against CYP-induced hemorrhagic cystitis and effectively improves bladder voiding dysfunction. Therefore, our results indicate that LUT may be a promising candidate for the prevention and treatment of hemorrhagic cystitis.

## Data Availability

The research data used to support the findings of this study are included within the article.
